# Multicenter real-world data on immunotherapy for R/M HNSCC from China: comprehensive analysis of efficacy and survival differences across diverse clinical backgrounds, and identification of predictive peripheral blood biomarkers

**DOI:** 10.3389/fimmu.2026.1720838

**Published:** 2026-02-04

**Authors:** Feifan Sun, Shasha Pu, Bingxin Hou, Jingyi Liu, Changhao Liu, Jian Zang, Haichuan Su, Zhongwei Wang, Xuejiao Song, Siyu Wang, Dongbo Jiang, Lina Zhao, Mei Shi

**Affiliations:** 1 Department of Radiation Oncology, Xijing Hospital, The Fourth Military Medical University (Air Force Medical University), Xi’an, China; 2 Department of Oncology, The General Hospital of Western Theater Command, Chengdu, China; 3 Department of Oncology, Tangdu Hospital, The Fourth Military Medical University (Air Force Medical University), Xi’an, China; 4 Department of Radiation Oncology, The Second Affiliated Hospital of Xi’an Jiaotong University (Xibei Hospital), Xi’an, China; 5 Department of Immunology, Basic Medicine School, The Fourth Military Medical University (Air Force Medical University), Xi’an, China

**Keywords:** efficacy and survival differences, immunotherapy, predictive markers, R/M HNSCC, real-world study

## Abstract

**Background:**

PD-1 inhibitors are first-line treatments for recurrent or metastatic head and neck squamous cell carcinoma (R/M HNSCC). However, previous trials included few participants from mainland China and other Asian regions and differed from real-world practice. PD-L1 expression alone has limited predictive value. This study aimed to systematically evaluate efficacy and survival differences in diverse clinical scenarios and identify associated peripheral blood markers (PBMs).

**Methods:**

Data were retrospectively collected from 105 R/M HNSCC patients treated with first-line immunotherapy alone or in combination across 3 hospitals in China (2020.01-2022.12). Primary endpoints were overall survival (OS) and progression-free survival (PFS). We assessed efficacy, survival, and safety, and developed predictive models. Data analyses were performed using SPSS 26.0 and GraphPad Prism 8.0.1.

**Results:**

The median follow-up was 21 months. The objective response rate was 37.14%. Median OS was 21 months (1-year OS: 81.02%), and median PFS was 11 months (1-year PFS: 39.47%). Immunotherapy was more effective for distant metastasis (DM) than local recurrence (LR). For patients with LR, DM, or both, median PFS was 12, 14, and 7.5 months, respectively (P = 0.0029). Higher combined positive score (CPS) predicted better outcomes. Median OS for CPS ≥ 20, 1 ≤ CPS < 20, and CPS < 1 was 32, 20, and 12 months, respectively (P = 0.0008). In the comparison of combination versus single-agent immunotherapy, median PFS was 12 versus 9 months (P = 0.0044). Combining taxanes with immunotherapy yielded favorable results, with a median OS of 27 months. No survival difference was found between domestic and imported PD-1 inhibitors. Secondary radiotherapy did not improve survival. Increased peripheral blood lymphocyte subsets and decreased peripheral blood inflammatory markers were associated with superior immunotherapy outcomes. Key predictive PBMs included baseline CD8^+^ T cells, CD3^+^ T cells at 12 weeks post-treatment (12w pt), CD4^+^ T cells at 6w pt, and CD8^+^ T cells at 12w pt.

**Conclusion:**

This study focused on evaluating immunotherapy efficacy and survival differences in R/M HNSCC patients across various clinical background. Dynamic PBMs correlated closely with immunotherapy response and survival prognosis. These findings expanded treatment options and supported personalized decisions. R/M HNSCC, Immunotherapy, Real-world study, Efficacy and survival differences, Predictive markers.

## 1 Introduction

Head and neck cancer is the seventh most common malignant tumor globally, with squamous cell carcinoma accounting for 90% of cases (1). Over 60% of patients present with locally advanced disease (stages III/IV, excluding M1). Despite multimodal treatments, 65% of these patients relapse or develop metastasis, and the 5-year survival rate for recurrent or metastatic head and neck squamous cell carcinoma (R/M HNSCC) is only 3.6% (2, 3). For most locally recurrent patients unsuitable for salvage surgery or re-irradiation, systemic therapy is preferred. The EXTREME regimen (cetuximab plus 5-fluorouracil and platinum) has been recommended as first-line treatment, but median overall survival (OS) remains at 10.1 months (1). Immune checkpoint inhibitors (ICI) have demonstrated improved survival in R/M HNSCC. PD-1 inhibitors are monoclonal antibodies that specifically block the interaction between PD-1 and PD-L1. This blockade alleviates T cell inhibition, reverses T cell exhaustion, and restores the ability of cytotoxic T cells to recognize and eliminate tumor cells (4). Data from CheckMate-141 and Keynote-040 showed Nivolumab and Pembrolizumab were second-line options after platinum-based chemotherapy (5, 6). The Keynote-048 established Pembrolizumab as a first-line treatment for R/M HNSCC. In patients with PD-L1 (combined positive score, CPS) ≥ 1%, both Pembrolizumab alone and combined with chemotherapy improved OS over the EXTREME regimen. Four-year follow-up showed long-term survival benefits in CPS ≥ 1 and CPS ≥ 20 patients (7).

However, randomized controlled trials (RCTs) often enroll highly selected patients, which differ substantially from real-world and lack broad representativeness (8, 9). The Keynote-048 study had several limitations compared to actual clinical practice: (1) Real-world treatment regimens are more complex, influenced by factors such as physical condition, economic status, and tolerability to side effects, leading to diverse drug combinations with ICI. (2) ICI options in the real world extend beyond imported drugs (Nivolumab and Pembrolizumab) to include domestic PD-1 inhibitors, requiring further validation of their efficacy. (3) Local recurrence (LR) accounts for 60-80% of cases in clinical practice but was underrepresented in Keynote-048 (27%), with LR patients showing poorer IT responses than metastatic patients. Lastly, previous clinical trials included limited representation from populations in mainland China and other Asian regions.

Despite the promise of PD-1 inhibitors for R/M HNSCC, only 13.3%-36.1% of patients benefit significantly from immunotherapy (10). PD-L1 expression remains the sole guideline-recommended biomarker for selecting immunotherapy candidates, but inconsistent study results question its reliability (11). Some patients with negative PD-L1 expression still benefit from ICI, highlighting the need for additional predictive markers (12, 13). Elevated peripheral blood inflammatory markers (PBIM), such as neutrophil-to-lymphocyte ratio (NLR) and platelet-to-lymphocyte ratio (PLR), have been associated with poor prognosis in ICI-treated patients and may predict treatment benefit (14–18). The role of peripheral blood lymphocyte subsets (PBLS) in predicting ICI efficacy and survival outcomes in R/M HNSCC requires further investigation (19, 20).

This multicenter real-world study aimed to comprehensively analyze the efficacy and survival differences among R/M HNSCC patients receiving ICI under various clinical scenarios. Additionally, it explored the dynamic changes in PBIM and PBLS over time to identify predictive biomarkers for immunotherapy benefits.

## 2 Materials and methods

### 2.1 Study design

**Figure 1:**
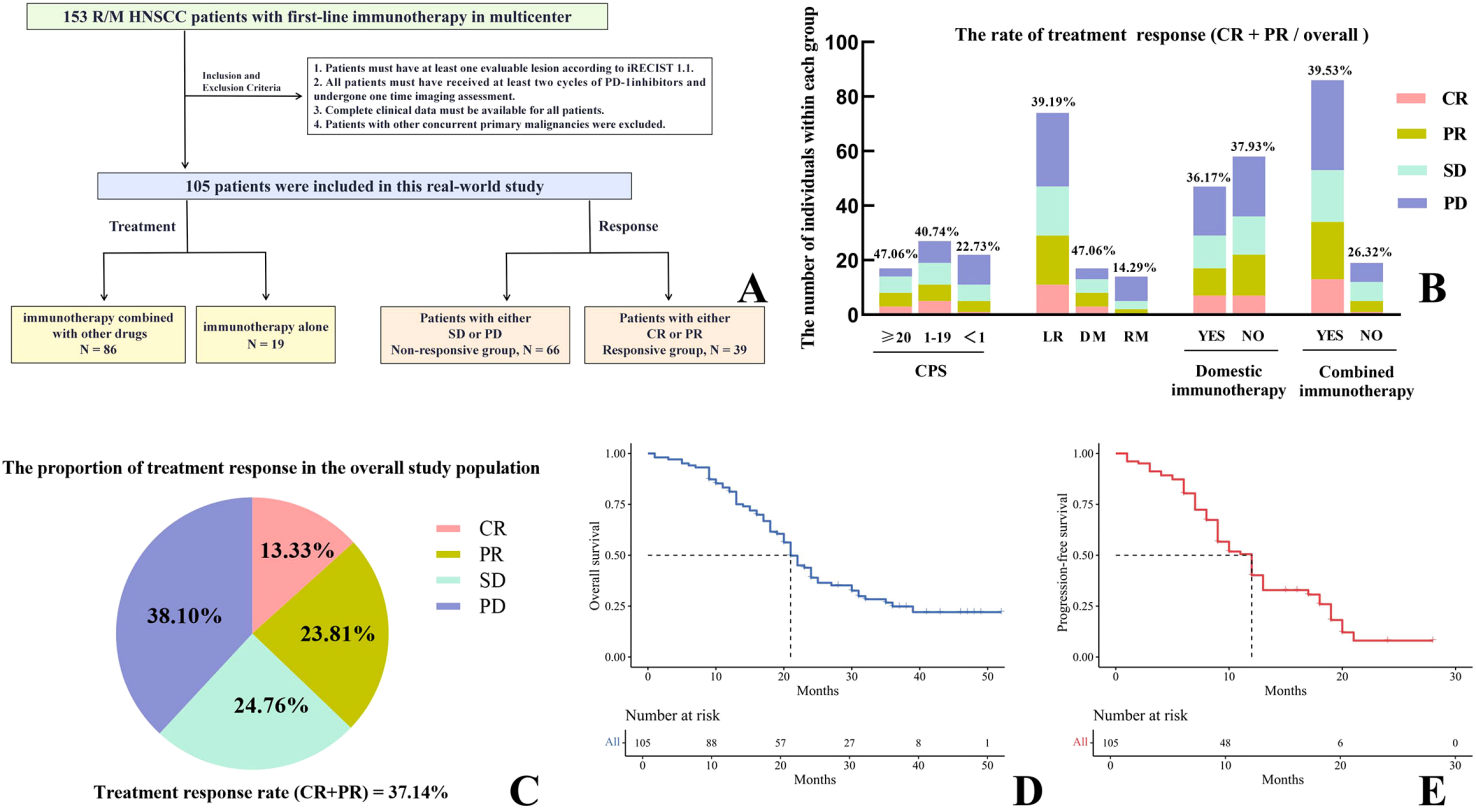
Overall design framework of the research **(A)**. Evaluation of therapeutic effect on the study population **(B, C)**. Survival analysis for the entire cohort **(D, E)**.

This multicenter retrospective real-world study analyzed clinical data, follow-up information, and peripheral blood markers (PBMs) from 153 R/M HNSCC patients treated with first-line PD-1 inhibitors (monotherapy or combination therapy) between January 2020 and December 2022 at three hospitals in Shaanxi Province, China (**Figure 1A**). After applying predefined inclusion and exclusion criteria, 105 eligible patients were included.

### 2.2 Patient selection

#### 2.2.1 Inclusion criteria

1. Patients aged ≥ 18 years with a pathological diagnosis of R/M HNSCC.
2. Initiation of first-line systemic therapy with PD-1 inhibitors (imported or domestic), administered as monotherapy or in combination with other agents.
3. Imaging assessments were performed after completion of at least two treatment cycles.
4. Presence of at least one measurable lesion according to the immune response evaluation criteria in solid tumors (iRECIST), version 1.1 (21).
5. Not eligible for local curative-intent therapy.
6. PBMs examination data must be available at baseline (prior to treatment), 6 weeks post-treatment (6w pt), and 12 weeks post-treatment (12w pt). It is important to clarify that the post-treatment time point in this context is defined from the initiation of the first immunotherapy cycle, rather than from the completion of the entire treatment course.

#### 2.2.2 Exclusion criteria

1. Diagnosis of nasopharyngeal carcinoma.
2. History of a second primary malignancy within the past five years.
3. Significant missing clinical or radiological data that would compromise the integrity and validity of the analysis.
4. Prior exposure to anti-PD-1 or anti-PD-L1 therapies.

### 2.3 Treatment regimen

PD-1 inhibitors were administered as monotherapy or in combination with chemotherapy (paclitaxel, gemcitabine, fluorouracil, platinum-based agents) or targeted drugs. PD-1 inhibitors include pembrolizumab (Merck & Co., Inc., USA), nivolumab (Bristol-Myers Squibb Holdings Pharma, Ltd., USA), toripalimab (Shanghai Junshi Bioscience Co., Ltd., China), tislelizumab (BeiGene, Ltd., China), zimberelimab (Guangzhou Yuheng Biotechnology Co., Ltd., China), and penpulimab (Zhengda Tianqing Kangfang [Shanghai] Biopharmaceutical Technology Co., Ltd., China). Treatment regimens were tailored based on patients’ physical condition, economic status, and tolerability to adverse effects. The regimen was repeated every three weeks. For patients achieving stable disease (SD), partial response (PR), or complete response (CR) following multiple cycles of treatment, PD-1 inhibitors were continued until disease progression or for a maximum duration of two years.

### 2.4 Data collection and follow-up

We collected the following information: name, gender, age, CPS, Eastern Cooperative Oncology Group Performance Status (ECOG PS), pathological findings, tumor location, clinical diagnosis and staging, treatment regimen, and treatment outcomes. We collected and analyzed the absolute values of PBLS and PBIM. These markers included total T cells (CD3^+^), helper T cells (CD4^+^), cytotoxic T cells (CD8^+^), total B cells (CD19 absolute count), natural killer (NK) cell counts, NLR, and PLR. Data were recorded via an electronic medical record system to ensure the protection of sensitive information, such as patient names and identification numbers.

For all patients, we conducted follow-ups every three months through telephone interviews or outpatient visits to collect data on treatment efficacy and adverse reactions. The collected data included OS, progression-free survival (PFS), and treatment-related adverse events (TRAEs). TRAEs were graded according to the Common Terminology Criteria for Adverse Events (CTCAE) version 5.0 (22), without distinguishing which specific drug caused the adverse events.

### 2.5 Study endpoints, efficacy assessment, and survival analysis

The primary endpoints of this study were PFS and OS. The secondary endpoints included the objective response rate (ORR), defined as the proportion of patients achieving CR or PR, and the disease control rate (DCR), defined as the proportion of patients achieving CR, PR, or SD, serving as efficacy measures. Patients with CR or PR were categorized as the responder group, whereas those with SD or progressive disease (PD) were classified as non-responder group.

PFS was defined as the time interval from the initiation of immunotherapy to the first documented evidence of disease progression or death from any cause. OS was defined as the time from the start of treatment to the date of patient death or last follow-up. Baseline measurements of all measurable lesions were obtained prior to treatment initiation, and regular imaging assessments were performed during follow-up.

All enrolled patients were stratified into subgroups based on various factors, including treatment regimens, patterns of disease progression, PD-L1 expression status, different primary tumor sites, and multiple PBMs. Survival outcomes and treatment efficacy were compared across these subgroups.

### 2.6 Statistical analysis and construction of survival risk prediction models

Data processing and graphical visualization were performed using SPSS 26.0 and GraphPad Prism 8.0.1 statistical software. Model construction was conducted using R software (version 4.3.2, https://www.r-project.org/). Descriptive statistics were employed to evaluate the clinical characteristics of patients, with the composition ratio indicating their specific classifications. Differences in PBMs between responders and non-responders were analyzed with PBMs treated as continuous variables. Inter-group differences for each biomarker at each follow-up time point were assessed. For normally distributed data, one-way ANOVA was employed for overall comparisons; if the result was statistically significant, *post hoc* pairwise comparisons were conducted using the independent samples t-test with Bonferroni adjustment. For non-normally distributed data, the Kruskal-Wallis H test was used for global comparisons, followed by Dunn’s test with Bonferroni correction for *post hoc* pairwise analyses. Survival curves were generated using the Kaplan-Meier method. Survival differences were compared using the Log-Rank test. Univariable Cox proportional hazards regression was used to assess the association between clinical variables and OS or PFS. To mitigate the risk of overfitting in the multivariate Cox proportional hazards model, we implemented a rigorous hierarchical variable selection approach. First, all clinicopathological variables were screened using univariate Cox regression analysis, with variables meeting a significance threshold of P < 0.05 retained for inclusion in multivariate modeling. Subsequently, backward stepwise elimination was performed based on the likelihood ratio test (removal criterion: P > 0.05) to ensure that only variables contributing independent and statistically significant prognostic value remained in the final model. The final multivariable model for PFS included six factors: age, sex, number of treatment cycles, treatment modality, ECOG performance status, and adverse reactions. The model for OS included three factors: number of treatment cycles, treatment modality, and hematotoxicity. The factor with a hazard ratio (HR) of 1 serves as the reference category. The proportional hazards assumption was verified using Schoenfeld residuals. All p-values and confidence intervals were determined via two-sided tests. A p-value < 0.05 was considered statistically significant.

The absolute values of NLR, PLR, and PBLS before and after treatment were treated as numerical variables to construct receiver operating characteristic (ROC) curves. The optimal cut-off values for NLR, PLR, and PBLS were determined based on the maximum Youden index (defined as sensitivity + specificity - 1) at baseline. Patients were stratified into high-level and low-level groups for survival analysis. The values at 6w and 12w pt were compared with the optimal cut-off values and categorized into decreased and increased groups for further survival analysis. The absolute values of dynamic changes in PBMs and survival information were integrated, and least absolute shrinkage and selection operator (LASSO) regression analysis was applied to select parameters with non-zero coefficients and obtain the optimal lambda value. Subsequently, a predictive model was established, and its performance was assessed using the area under the curve (AUC). Finally, a nomogram was constructed by integrating the LASSO model and clinical characteristics to evaluate survival benefit following ICI in R/M HNSCC patients.

## 3 Results

### 3.1 Clinical characteristics of research subjects

**Table 1** summarized the baseline clinical characteristics of 105 patients with R/M HNSCC. Among them, 82 patients (78.09%) were younger than 65 years, with a median age of 58 years (range: 26–84 years). The cohort included 78 males (74.29%). ECOG PS was 0 in 26.67%, 1 in 68.57%, and 2 in 4.76% of patients. Primary tumor sites were distributed as follows: oropharynx (44.76%), oral cavity (40.95%), larynx (6.67%), hypopharynx (4.76%), and nasal cavity/sinuses (2.86%). PD-L1 CPS distribution was as follows: < 1 (16.19%), 1-19 (25.71%), ≥ 20 (20.95%), and unknown (37.15%). A further comparative analysis of the baseline characteristics revealed relatively balanced distributions between the populations with known and unknown CPS (**Supplementary Table 1**). Regarding to disease pattern, 70.47% of patients had local recurrence only (LR), 16.19% had distant metastasis only (DM), and 13.34% had both local recurrence and distant metastasis (RM).

**Table 1:** The baseline characteristics of 105 cases of R/M HNSCC patients.

Characteristics	Cases (n)	Proportion (%)
Age (year)		
≥ 65	23	21.91%
<65	82	78.09%
Gender		
Female	27	25.71%
Male	78	74.29%
Primary Site		
Nasal cavity/sinuses	3	2.86%
oral cavity	43	40.95%
oropharynx	47	44.76%
Hypopharyngeal	5	4.76%
Larynx	7	6.67%
ECOG Score		
0	28	26.67%
1	72	68.57%
2	5	4.76%
CPS Score		
<1	17	16.19%
≥ 1,<20	27	25.71%
≥ 20	22	20.95%
Unkow	39	37.15%
Recurrence/Metastasis		
Local Recurrence Only	74	70.47%
Distant Metastasis Only	17	16.19%
Local Recurrence and Distant Metastasis	14	13.34%

### 3.2 Key features of treatment strategies

Treatment regimens for the 105 patients were summarized in **Table 2**. The median number of ICI cycles administered was five. A total of 55.24% of patients received imported PD-1 inhibitors (pembrolizumab: n = 54; nivolumab: n = 4), while 44.76% received domestically developed PD-1 inhibitors (toripalimab: n = 25; tislelizumab: n = 17; zimberelimab: n = 4; penpulimab: n = 1). Combination therapy was administered to 86 patients (81.90%), consisting of immunotherapy combined with targeted agents (anlotinib: n = 3; nimotuzumab: n = 6) or chemotherapy (single-agent: n = 6; dual-agent: n = 68; triple-agent: n = 3). Chemotherapy regimens were predominantly taxane-based (n = 48) or gemcitabine-based (n = 17), with only three patients receiving fluorouracil in combination with platinum-based chemotherapy. Secondary radiotherapy was delivered in 12.38% of patients, including re-irradiation for LR and palliative radiotherapy for DM.

**Table 2:** The treatment characteristics of 105 cases of R/M HNSCC patients.

Medicines	Cases (n)	Proportion (%)
PD-1 Inhibitors	105	100%
Pembrolizumab	54	51.43%
Nivolumab	4	3.81%
Toripalimab	25	23.81%
Tislelizumab	17	16.19%
Zimberelimab	4	3.81%
Penpulimab	1	0.95%
Combined Medicines	86	81.90%
Paclitaxel-albumin + cispatin	29	33.72%
Gemcitabine + cispatin	14	16.28%
Docetaxel + cispatin	2	2.32%
Paclitaxel-albumin + nedaplatin	9	10.46%
Gemcitabine + nedaplatin	3	3.49%
Anlotinib	3	3.49%
Capecitabine	3	3.49%
Nimotuzumab	6	6.98%
S-1	3	3.49%
Paclitaxel-albumin	8	9.30%
Capecitabine + cispatin	3	3.49%
Paclitaxel-albumin + nedaplatin + 5-FU	3	3.49%
Subsequent Radiotherapy		
Yes	13	12.38%
No	92	87.62%

### 3.3 Efficacy evaluation and survival analysis across varied clinical background

#### 3.3.1 Overall survival analysis and efficacy assessment in the study population

The median follow-up duration was 21 months. Among the patients, 13.33% achieved CR, 23.81% achieved PR, 24.76% had SD, and 38.10% experienced PD. The ORR was 37.14%, and the DCR was 61.90% (**Figure 1C**). As shown in **Figure 1B**, subgroup analyses revealed differences in ORR according to CPS levels (≥ 20: 47.06%; 1-19: 40.74%; < 1: 22.73%), recurrence patterns (LR: 39.19%; DM: 47.06%; RM: 14.29%), type of PD-1 inhibitor (domestic: 36.17%; imported: 37.93%), and immunotherapy regimen (combination therapy: 39.53%; monotherapy: 26.32%). The median PFS was 11 months (95% CI: 9-13) (**Figure 1E**), and the median OS was 21 months (95% CI: 20-24) (**Figure 1D**). At 12 months, the PFS rate was 39.47% (95% CI: 30.39%-51.26%) and the OS rate was 81.02% (95% CI: 73.69%-89.09%). At 24 months, the OS rate was 38.30% (95% CI: 29.46%-49.78%), with no PFS events observed.

#### 3.3.2 Higher CPS values were associated with improved prognosis

**Figure 2:**
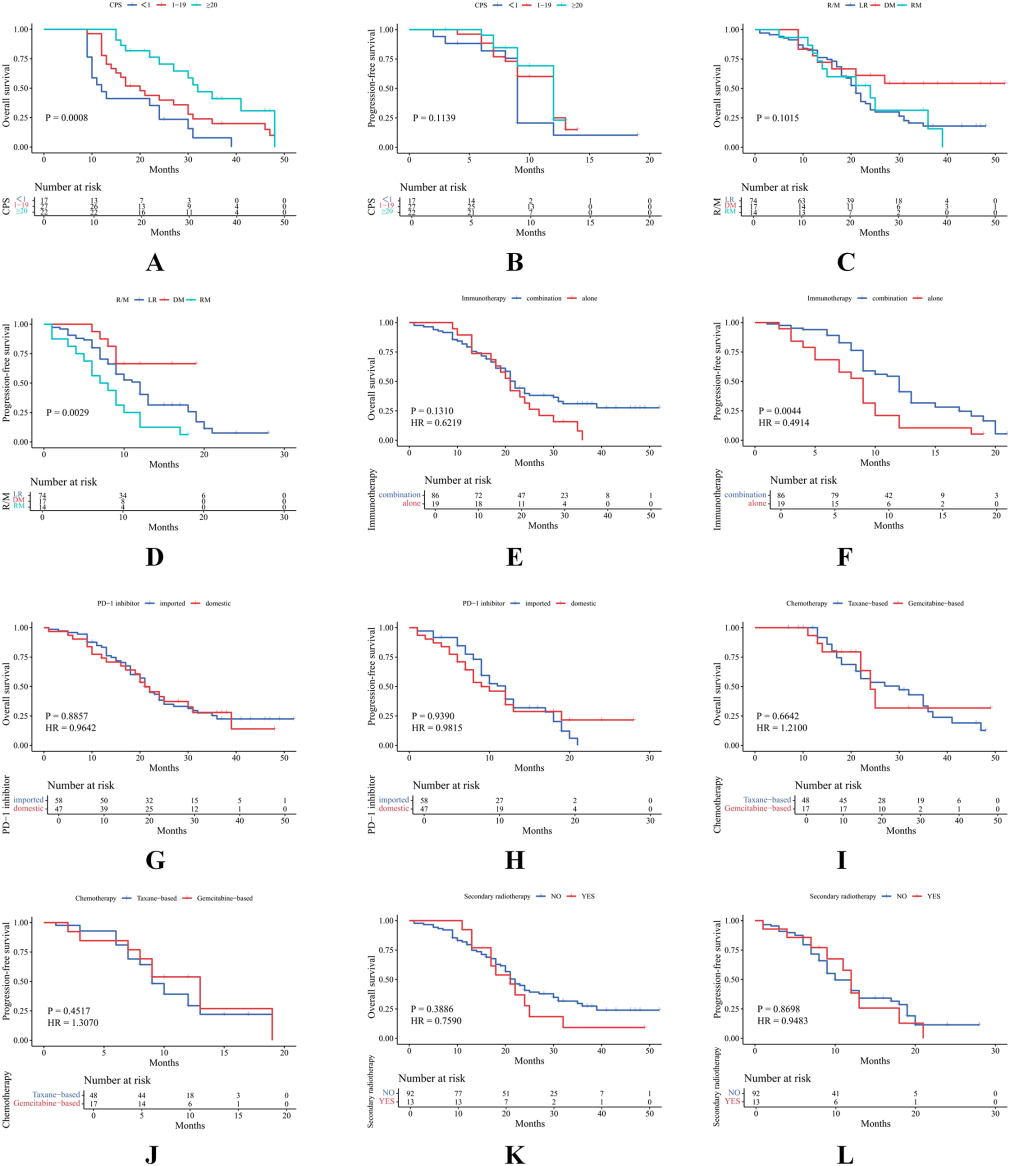
Survival analysis in different clinical scenarios. **(A, B)** Comparison of OS and PFS among patients with CPS ≥ 20, 1 ≤ CPS < 20, and CPS < 1. **(C, D)** Comparison of OS and PFS among patients with LR, DM, and RM. **(E, F)** Comparison of OS and PFS between combination immunotherapy and immunotherapy alone. **(G, H)** Comparison of OS and PFS between imported and domestic PD-1inhibitors. **(I, J)** Comparison of OS and PFS between taxane-based and gemcitabine -based chemotherapy. **(K, L)** Comparison of OS and PFS between patients with and without secondary radiotherapy.

For patients with CPS ≥ 20, 1 ≤ CPS < 20, and CPS < 1, median OS was 32, 20, and 12 months, respectively (P = 0.0008), and median PFS was 12, 12, and 9 months, respectively (P = 0.1139) (**Figures 2A, B**). When comparing CPS ≥ 20 versus CPS < 1, the difference in median OS was statistically significant (32 vs. 12 months, HR = 0.29, P = 0.0002), as was PFS (12 vs. 9 months, HR = 0.50, P = 0.0439) (**Supplementary Figures 1A, B**). Given that 37.15% of the CPS data were missing, we performed a sensitivity analysis and applied multiple imputation to address the incomplete data. The results further supported an association between higher CPS scores and improved survival outcomes (**Supplementary Figures 2-4**).

(3) ICI demonstrated greater efficacy in patients with DM compared to those with LR.

Among patients with LR, DM, and RM, median OS was 21, 23, and 21 months (P = 0.1015), and median PFS was 12, 14, and 7.5 months (P = 0.0029) (**Figures 2C, D**). Combination immunotherapy was associated with significantly longer PFS than monotherapy (12 vs. 9 months, HR = 0.49, P = 0.0044), although no significant difference in OS was observed (22 vs. 21 months, P = 0.1310) (**Figures 2E, F**). However, among patients with LR, no significant differences in OS or PFS were observed between combination therapy and monotherapy (**Supplementary Figures 1C, D**).

#### 3.3.3 No survival difference between domestic and imported PD-1 inhibitors

No significant differences in survival outcomes were found between domestic and imported PD-1 inhibitors (OS: 21 vs. 21 months, P = 0.8857; PFS: 12 vs. 9 months, P = 0.9390) (**Figures 2G, H**).

#### 3.3.4 Adding taxanes to ICI showed favorable trends, but secondary radiotherapy did not improve survival

Furthermore, no significant differences were observed between taxane-based and gemcitabine-based chemotherapy regimens (OS: 27 vs. 24 months, P = 0.6642; PFS: 9 vs. 13 months, P = 0.4517) or between patients who received versus those who did not receive secondary radiotherapy (OS: 22 vs. 21 months, P = 0.3886; PFS: 10 vs. 12 months, P = 0.8698) (**Figures 2I**-**L**).

#### 3.3.5 Patients with hypopharyngeal or nasal cavity/sinus tumors exhibited shorter PFS

Across primary tumor sites—including oral cavity, oropharynx, hypopharynx, larynx, and nasal cavity/sinus—median OS was 24, 18, 26.5, 23, and 22 months (P = 0.4254), respectively, while median PFS was 12, 10, 6.5, 18, and 6 months (P = 0.0328) (**Supplementary Figures 1E, F**).

### 3.4 Analysis of risk factors and treatment-related adverse events

Univariate and multivariate Cox regression analyses were conducted to identify risk factors associated with patient outcomes. Female sex (HR = 0.543, P = 0.019), receipt of ≥ 5 treatment cycles (HR = 0.401, P = 0.012), combination immunotherapy (HR = 0.372, P = 0.002), ECOG 0 (vs. ECOG 1: HR = 3.117, P = 0.010; vs. ECOG 2: HR = 9.246, P < 0.0001), and occurrence of grade 1–2 TRAEs (HR = 8.724, P = 0.0007) were significantly associated with improved PFS (**Supplementary Table 2**). In contrast, ≥ 5 treatment cycles correlated with worse OS (HR = 2.388, P = 0.035) (**Supplementary Table 3**). This discrepancy may be attributed to the development of TRAEs. As shown in **Supplementary Figure 5**, the number of treatment cycles exhibited a positive correlation with both the severity of adverse reactions (correlation coefficient: 0.2188) and PFS (correlation coefficient: 0.0984), but a negative correlation with OS (correlation coefficient: -0.0032). In addition, the number of treatment cycles was associated with therapeutic efficacy. Patients receiving ≥ 5 cycles achieved an ORR of 43.63% and a DCR of 69.09%, both higher than those observed in patients receiving < 5 cycles (**Supplementary Table 4**). These findings provided potential explanations for the improved PFS observed in patients receiving ≥ 5 cycles, despite the lack of corresponding improvement in OS. Grade 1–2 TRAEs occurred in 47.62% of patients, and ≥ grade 3 TRAEs were reported in 9.52% (**Supplementary Table 5**).

### 3.5 Higher PBLS and lower PBIM levels were associated with improved efficacy

**Figure 3:**
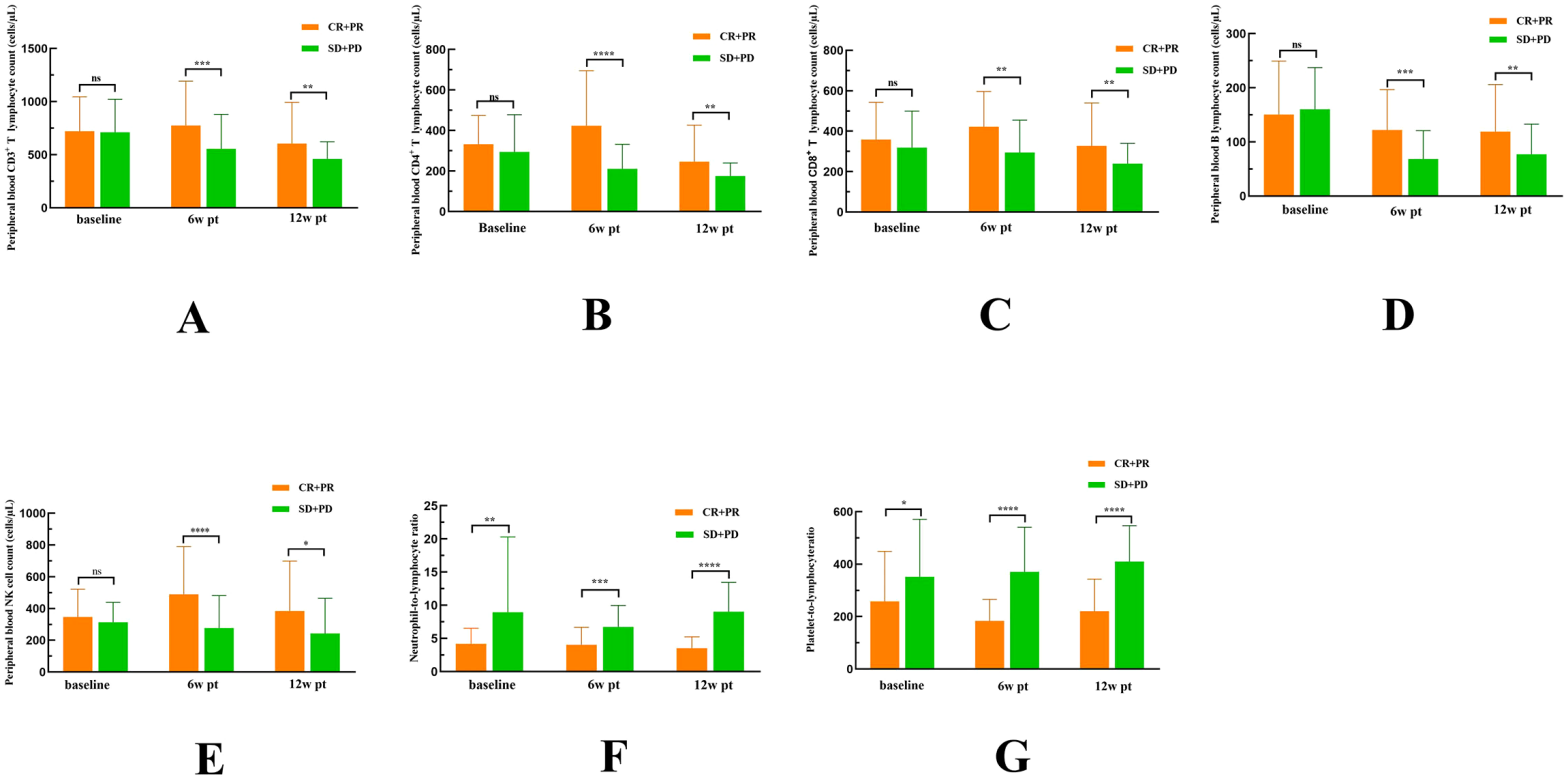
Comparison of dynamic changes in CD3^+^ T cells **(A)**, CD4^+^ T cells **(B)**, CD8^+^ T cells **(C)**, B cells **(D)**, NK cells **(E)**, NLR **(F)**, and PLR **(G)** at baseline, 6w pt, and 12w pt between immunotherapy responders and non-responders.

Given the delayed onset of immunotherapy effects, clinical responses are typically observed after 3 to 4 treatment cycles. Therefore, we retrospectively collected PBMs data at three time points. At baseline, NLR (P = 0.0052) and PLR (P = 0.0341) were significantly lower in the responder group compared to the non-responder group, whereas PBLS levels did not differ significantly between the two groups. At 6w pt, the responder group exhibited significantly higher levels of CD3^+^ T cells (P = 0.0003), CD4^+^ T cells (P < 0.0001), CD8^+^ T cells (P = 0.0098), B cells (P = 0.0003), and NK cells (P < 0.0001), along with significantly lower NLR (P = 0.0002) and PLR (P < 0.0001). Similarly, at 12w pt, the responder group showed elevated levels of CD3^+^ T cells (P = 0.0077), CD4^+^ T cells (P = 0.0042), CD8^+^ T cells (P = 0.0055), B cells (P = 0.0052), and NK cells (P = 0.0111), as well as markedly reduced NLR (P < 0.0001) and PLR (P < 0.0001). These findings were presented in **Figure 3**.

### 3.6 Dynamic CD3^+^, CD4^+^, CD8^+^, NLR, and PLR in peripheral blood significantly linked to survival

**Figure 4:**
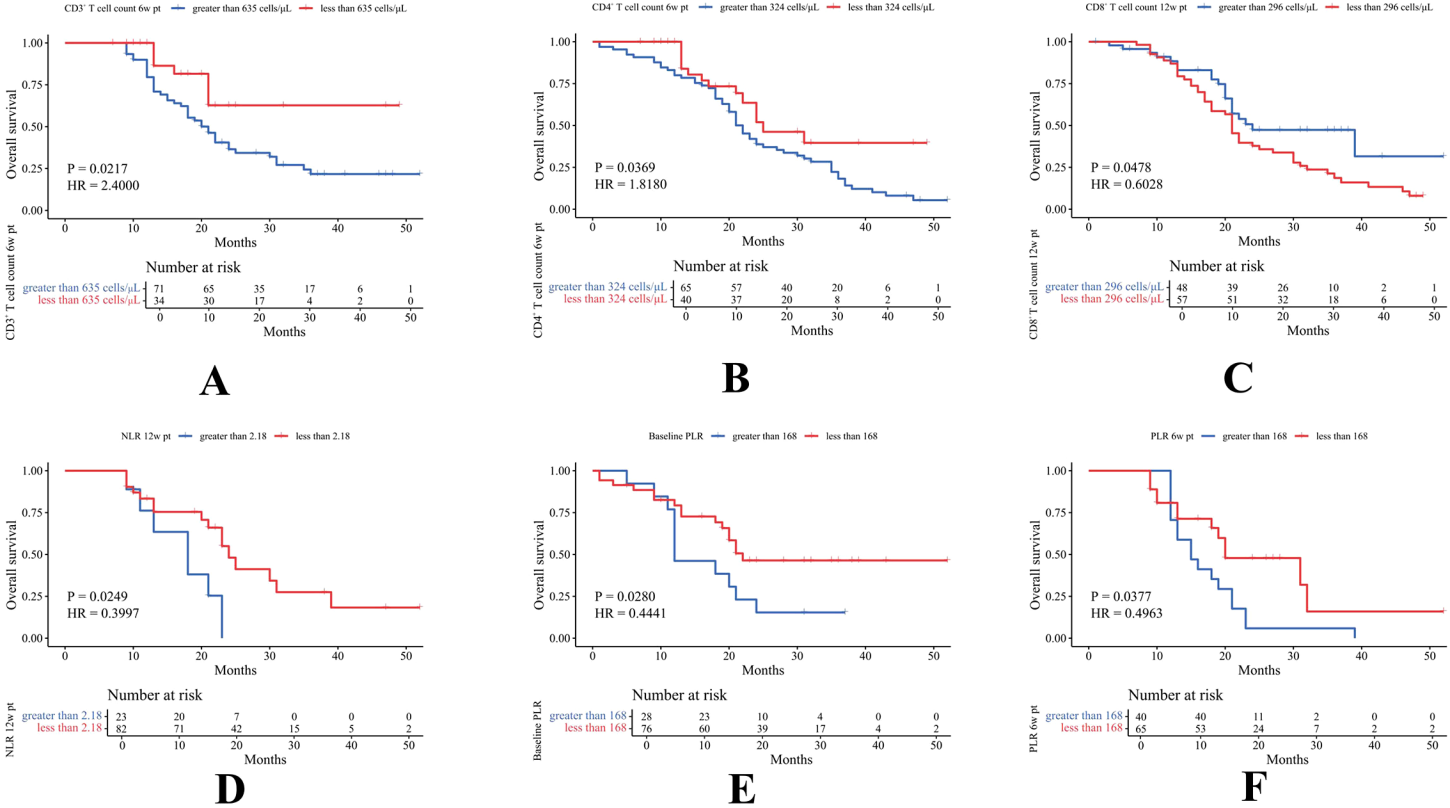
According to OS-based cutoff values, marker expression was categorized into two groups, and statistically differences were observed in CD3^+^ 6w pt **(A)**, CD4^+^ 6w pt **(B)**, CD8^+^ 12w pt **(C)**, NLR 12w pt **(D)**, baseline PLR **(E)**, and PLR 6w pt **(F)**.

**Figure 5:**
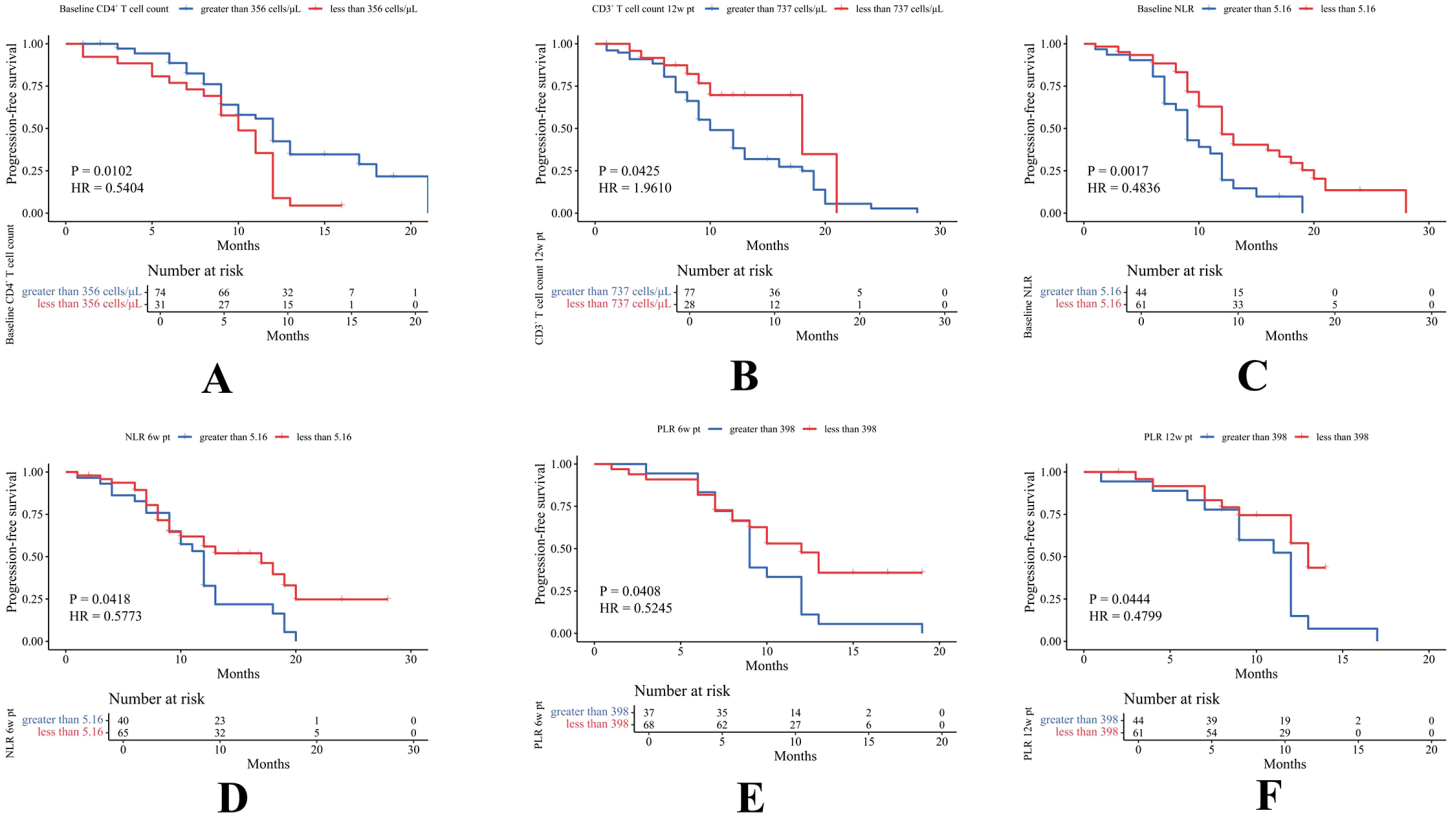
According to PFS-based cutoff values, marker expression was categorized into two groups, and statistically differences were observed in baseline CD4^+^**(A)**, CD3^+^ 12w pt **(B)**, baseline NLR **(C)**, NLR 6w pt **(D)**, PLR 6w pt **(E)**, and PLR 12w pt **(F)**.

The optimal cutoff values for PBMs based on OS were as follows: B cells, 96 cells/μL; CD3^+^ T cells, 635 cells/μL; CD4^+^ T cells, 324 cells/μL; CD8^+^ T cells, 296 cells/μL; NK cells, 76 cells/μL; NLR, 2.18; and PLR, 168. Those based on PFS were: B cells, 91 cells/μL; CD3^+^ T cells, 737 cells/μL; CD4^+^ T cells, 356 cells/μL; CD8^+^ T cells, 354 cells/μL; NK cells, 89 cells/μL; NLR, 5.16; and PLR, 398. PBMs were subsequently categorized into up and down expression groups according to their respective cutoff values. Key findings revealed that poorer OS was significantly associated with elevated levels of CD3^+^ T cells (HR = 2.40, P = 0.0217) at 6w pt, CD4^+^ T cells (HR = 1.82, P = 0.0369) at 6w pt, PLR at baseline (HR = 0.44, P = 0.0280), PLR at 6w pt (HR = 0.50, P = 0.0377), NLR at 12w pt (HR = 0.40, P = 0.0249), and reduced CD8^+^ T cell levels at 12w pt (HR = 0.60, P = 0.0478) (**Figure 4**). Similarly, worse PFS was significantly associated with elevated CD3^+^ T cells at 12w pt (HR = 1.96, P = 0.0425), NLR at baseline (HR = 0.48, P = 0.0017), NLR at 6w pt (HR = 0.58, P = 0.0418), PLR at 6w pt (HR = 0.52, P = 0.0408), PLR at 12w pt (HR = 0.48, P = 0.0444), and reduced CD4^+^ T cell levels at baseline (HR = 0.54, P = 0.0102) (**Figure 5**). Survival analyses for the remaining PBMs, which did not reach statistical significance, were presented in **Supplementary Figure 6**.

### 3.7 Development of a survival prediction model

**Figure 6:**
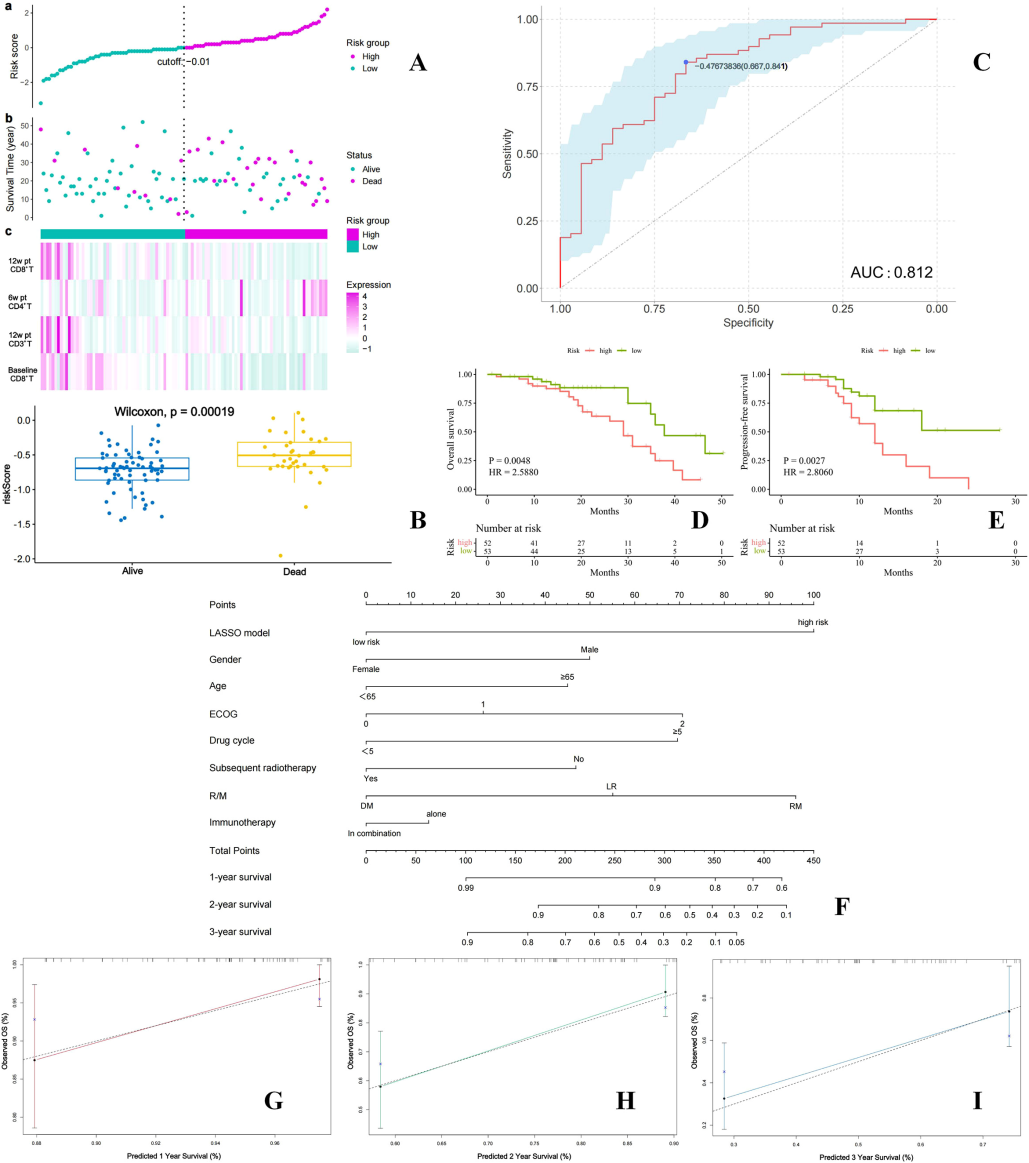
Development of a LASSO regression-based survival risk prediction model: Stratification of patients into high and low risk groups based on risk scores **(A)**; Comparison of risk scores between surviving and deceased patients **(B)**; ROC curve of the model **(C)**; Comparison of OS and PFS between high and low risk groups **(D, E)**. A nomogram for comprehensive prognostic evaluation **(F)**; **(G–I)** Calibration curves for one-Year, two-Year, and three-Year survival rates in the nomogram.

Based on the findings presented above, we developed a survival prediction model for patients with R/M HNSCC receiving immunotherapy. By integrating survival data with dynamic PBMs and applying LASSO regression, we constructed a robust model incorporating four key variables: baseline CD8^+^ T cells, CD3^+^ T cells at 12w pt, CD4^+^ T cells at 6w pt, and CD8^+^ T cells at 12w pt (**Supplementary Figure 7**). The model demonstrated strong discriminative ability, achieving an AUC of 0.812 (95% CI: 0.725-0.899) for predicting post-immunotherapy survival (**Figure 6C**). Internal validation revealed significantly higher risk scores in deceased patients compared to survivors (P = 0.0002) (**Figure 6B**). Using this model, 105 patients were stratified into high-risk and low-risk groups (**Figure 6A**), with the high-risk group showing markedly worse OS (HR = 2.59, P = 0.0048) and PFS (HR = 2.81, P = 0.0027) (**Figures 6D, E**). Furthermore, by combining the model with clinical prognostic factors, we developed a nomogram to facilitate individualized prognostic assessment (**Figure 6F**), which achieved a C-index of 0.841. Calibration curves indicated good agreement between predicted and observed 1-year, 2-year, and 3-year survival probabilities (**Figures 6G–I**). These results collectively confirmed the model’s high predictive accuracy and clinical utility.

## 4 Discussion

Currently, there is a critical need for additional clinical studies from diverse regions to determine the optimal treatment regimen for immunotherapy in R/M HNSCC. This real-world study considered various clinical contexts, including physical condition, disease status, economic circumstances, drug tolerance, and combination therapy regimens. It also identified predictive PBMs associated with immunotherapy benefits in these patients.

The ORR of this study was comparable to Keynote-048. Additionally, we also confirmed that patients with CPS ≥ 1, particularly CPS ≥ 20, as well as those receiving combination therapy instead of ICI monotherapy, demonstrated more pronounced survival benefits (7). However, 37.15% of the patients in this study lacked CPS data, a limitation closely tied to real-world challenges in the accessibility, cost, and standardization of PD-L1 expression testing. This disparity underscored a fundamental distinction between RCTs and real-world evidence. RCTs establish efficacy under controlled and ideal conditions, while real-world studies assess effectiveness amid practical constraints. To mitigate potential bias arising from missing data, we performed sensitivity analyses and applied multiple imputation, yielding results consistent with our primary findings. The study identified three cases of nasal cavity/sinus cancer. All received immunotherapy combined with chemotherapy (ICCT), resulting in one CR, one PR, and one PD. Five patients with ECOG 2 were included; four received ICCT and one received IT alone, with outcomes of one CR, two PR, and two PD. PFS for nasal cavity/sinus cancer and hypopharyngeal cancer was poorer compared to other HNSCC subtypes. Given the limited number of ECOG 2 patients, no detailed survival comparison was conducted between them and ECOG 0–1 patients. The proportion of LR patients (70.47%) more closely reflected clinical reality compared to Keynote-048 (27% LR). DM patients exhibited better PFS than LR or RM patients. No significant difference in OS or PFS was observed between ICI combination and alone in LR patients, suggesting distant metastatic lesions respond better to systemic treatment.

PD-1 inhibitors included both imported and domestic agents, while combination therapy regimens encompassed chemotherapy and targeted drugs in this study. Primary chemotherapy regimens were based on taxanes, platinum, and gemcitabine. Only 12 patients received fluorouracil-based drugs. Significant differences were observed in treatment processes compared to Keynote-048, particularly in combination chemotherapy regimens involving 5-fluorouracil and platinum-based drugs. No significant difference in survival or efficacy was observed between domestic and imported PD-1 inhibitors, findings consistent with prior reports (23, 24). Recent studies validated the efficacy and safety of paclitaxel-based and gemcitabine-based drugs in HNSCC (25–27). No significant difference was found between taxane-based and gemcitabine-based chemotherapy in this study. Due to the limited number of patients receiving fluorouracil-based drugs, no further analysis was conducted. A case report has confirmed the abscopal effect in R/M HNSCC receiving immunotherapy combined with radiotherapy (IRT) (28). However, our study found no significant differences in PFS or OS between patients who received IRT and those who did not. Similarly, a Phase II trial (NCT02684253) comparing nivolumab monotherapy with nivolumab plus radiotherapy in R/M HNSCC failed to demonstrate an abscopal effect or improved treatment response (29). Multiple IRT trials are currently ongoing to optimize the combination of ICI and radiotherapy (30).

The high proportion of patients (81.90%) receiving combination immunotherapy may explain why the median OS and PFS in this study exceeded those in Keynote-048. A U.S. real-world study of 83 R/M HNSCC patients showed a median OS of 14.9 months with first-line pembrolizumab combined with platinum and taxane drugs (31). An Austrian study reported a median OS of 21.3 months for 22 R/M HNSCC patients treated with first-line pembrolizumab plus docetaxel (32). A Chinese real-world study (23) and KEYNOTE-B10 (26) also supported the use of PD-1 inhibitors combined with taxanes-based chemotherapy in R/M HNSCC. Our research suggested that this combination had the potential to replace or serve as an alternative to the Keynote-048 treatment model, especially for patient intolerant to fluorouracil-based chemotherapy.

Although we have known that CPS-positive patients derive clinical benefit from immunotherapy, the ORR in CPS ≥ 20 patients following ICCT remains limited at 43.7% (33). Studies indicate that CPS-negative patients can still achieve clinical benefits from immunotherapy (34). No single biomarker reliably predicts immunotherapy efficacy (35). PBLS, particularly CD8^+^ T cells, are strongly associated with improved prognosis in HNSCC patients (36, 37). Additionally, prior studies have highlighted that NLR and PLR are linked to poor prognosis and elevated risk in patients with various types of malignancies (38, 39). This study confirmed that the dynamic changes in the peripheral blood immune environment were important suggestions of the efficacy and survival prognosis of immunotherapy in R/M HNSCC patients. We observed significant increases in peripheral blood levels of CD3^+^ T cells, CD4^+^ T cells, CD8^+^ T cells, and NK cells in responders at 6 and 12 weeks after initial treatment. Concurrently, the systemic inflammatory markers (NLR, PLR) showed decreases at these time points compared with non-responders. The early expansion of peripheral effector T cells during treatment suggests that ICI successfully reinvigorate exhausted T cells and promote the activation of tumor antigen-specific immune responses (37). The decline in NLR and PLR suggest that the treatment effectively controls tumor-related systemic inflammation, which is known to suppress anti-tumor immunity and promote disease progression (16, 38). Therefore, dynamic monitoring of these accessible blood indicators provides an opportunity for real-time assessment of the immune activation status in the body.

Despite being the guideline-recommended predictive biomarker, PD-L1 CPS faces several challenges in real-world application. First, it requires tumor tissue samples, which may be difficult to re-obtain in patients with recurrent or metastatic disease. Second, results exhibit heterogeneity due to variations in testing platforms, antibody clones, and interpretation criteria. Third, as a static measurement, it fails to capture the dynamic immune changes induced by treatment. Furthermore, although predictive models based on multi-omics or complex radiomics show promise, their clinical translation is limited by high costs, reliance on specialized infrastructure, and prolonged turnaround times. These barriers hinder routine implementation, especially in resource-constrained settings (35). In contrast, PBMs provide key practical advantages, including easy accessibility for serial monitoring, compatibility with standardized laboratory assays, and lower costs. This study demonstrated that dynamic PBMs offered prognostic information independent of PD-L1 CPS. Unlike static PD-L1, dynamic PBMs capture the evolving systemic immune response to treatment. For instance, even patients with negative CPS could experience survival benefit if they exhibited a post-treatment increase in CD8^+^ T cells and a decrease in NLR. Based on these findings, we developed a survival prediction model integrating dynamic PBMs. Key model variables—baseline CD8^+^ T cells, CD3^+^ and CD8^+^ T cells at 12 w pt, and CD4^+^ T cells at 6 w pt—carried clear biological interpretations. Baseline CD8^+^ T cells may represent a pre-existing anti-tumor immune reserve capable of reinvigoration, while the early sustained increase in circulating T cells (6–12 weeks) confirms effective treatment-induced immune activation. This model translated complex immune dynamics into a quantifiable, individual risk score, providing a practical tool to aid clinical decision-making.

This study shared limitations common in real-world research, including a relatively short median follow-up duration of 21 months and unavailable CPS for 39 patients. Future investigations should incorporate larger-scale, multi-center datasets with extended follow-up durations to validate these findings.

## 5 Conclusion

This study aimed to evaluate differences in efficacy and survival among patients with R/M HNSCC receiving immunotherapy across diverse clinical settings, and to identify PBMs associated with prognosis. Patients with CPS ≥ 20 demonstrated improved clinical outcomes. Immunotherapy was more effective in patients with DM than in those with LR. No survival differences were observed between domestically produced and imported PD-1 inhibitors. The combination of taxane-based regimens with immunotherapy yielded favorable therapeutic responses. Secondary radiotherapy did not confer a survival advantage. PBMs showed strong predictive value for survival risk stratification. This study provided valuable insights for clinical practice, expanded available treatment options, and supported personalized decision-making and treatment optimization.

## Data Availability

The original contributions presented in the study are included in the article/**Supplementary Material**. Further inquiries can be directed to the corresponding author/s.
