# Supplementary material for: Multicenter real-world data on immunotherapy for R/M HNSCC from China: comprehensive analysis of efficacy and survival differences across diverse clinical backgrounds, and identification of predictive peripheral blood biomarkers
Source: Front Immunol. 2026 Feb 4;17:1720838. doi: 10.3389/fimmu.2026.1720838 (PMC12913446; doi:10.3389/fimmu.2026.1720838)

Supplementary Table 1. Comparison of clinical characteristics between patients with known and unknown CPS.

| **Characteristics** | **Known CPS**  **(n = 66)** | **Unknown CPS**  **(n = 39)** |
| --- | --- | --- |
| **Age (year)** |  |  |
| ≥ 65 | 14 (21.21%) | 9 (23.08%) |
| ＜65 | 52 (78.79%) | 30 (76.92%) |
| **Gender** |  |  |
| Female | 17 (25.76%) | 10 (25.64%) |
| Male | 49 (74.24%) | 29 (74.36%) |
| **ECOG Score** |  |  |
| 0 | 17 (25.75%) | 11 (28.21%) |
| 1 | 46 (69.70%) | 26 (66.67%) |
| 2 | 3 (4.55%) | 2 (5.12%) |
| **Recurrence / Metastasis** |  |  |
| Local Recurrence Only | 47 (71.21%) | 27 (69.23%) |
| Distant Metastasis Only | 10 (15.15%) | 7 (17.95%) |
| Local Recurrence and Distant Metastasis | 9 (13.64%) | 5 (12.82%) |
| **Treatment regimen** |  |  |
| Combined immunotherapy | 55 (83.33%) | 31 (79.49%) |
| Immunotherapy monotherapy | 11 (16.67%) | 8 (20.51%) |
| **PD-1 inhibitor** |  |  |
| Imported drug | 37 (56.06%) | 21 (53.85%) |
| Domestic drug | 29 (43.94%) | 18 (46.15%) |

Supplementary Figure 1. Survival analysis in different clinical situations. A B: The comparison of OS and PFS between patients with CPS ≥ 20 and those with CPS < 1; C D: The comparison of OS and PFS between combination immunotherapy and immunotherapy alone in patients with only local recurrence; E F: The comparison of OS and PFS among different primary tumor sites.


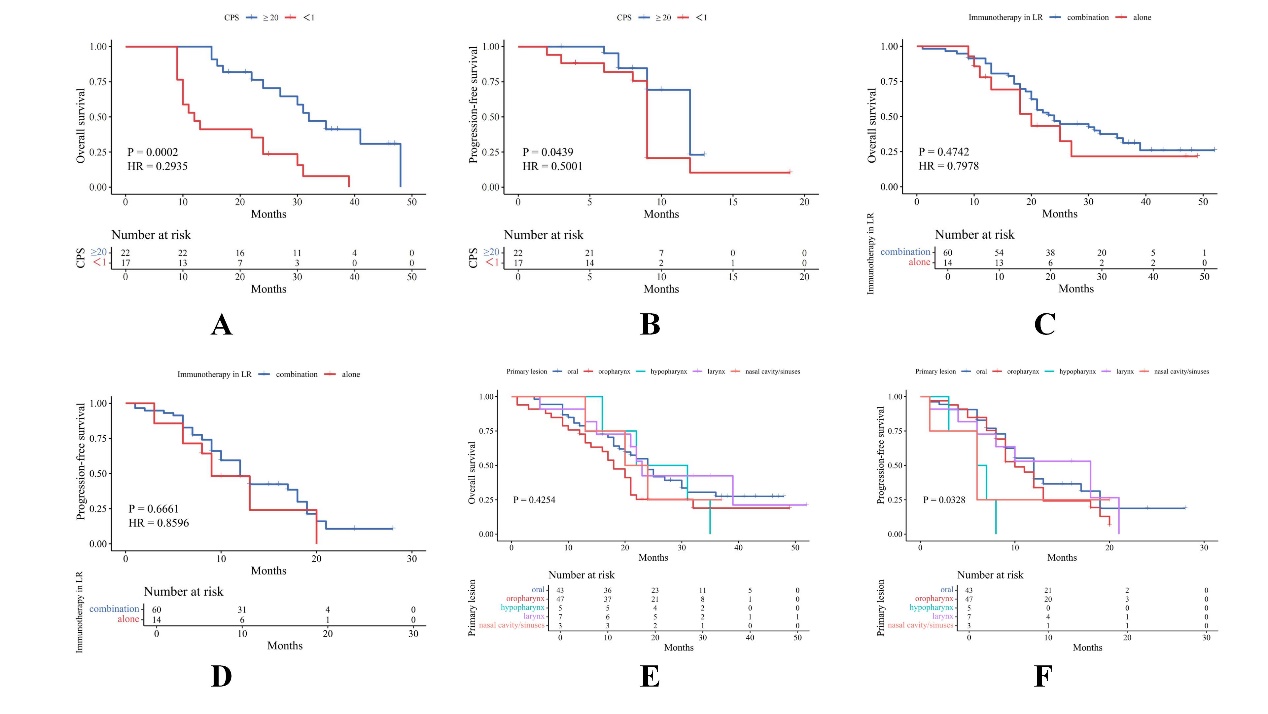


Supplementary Figure 2. Sensitivity analysis was performed by assigning patients with unknown CPS values to each of the three subgroups (CPS < 1, 1 ≤ CPS < 20, and CPS ≥ 20) separately, followed by re-evaluation of survival, including OS (A B C) and PFS (D E F).


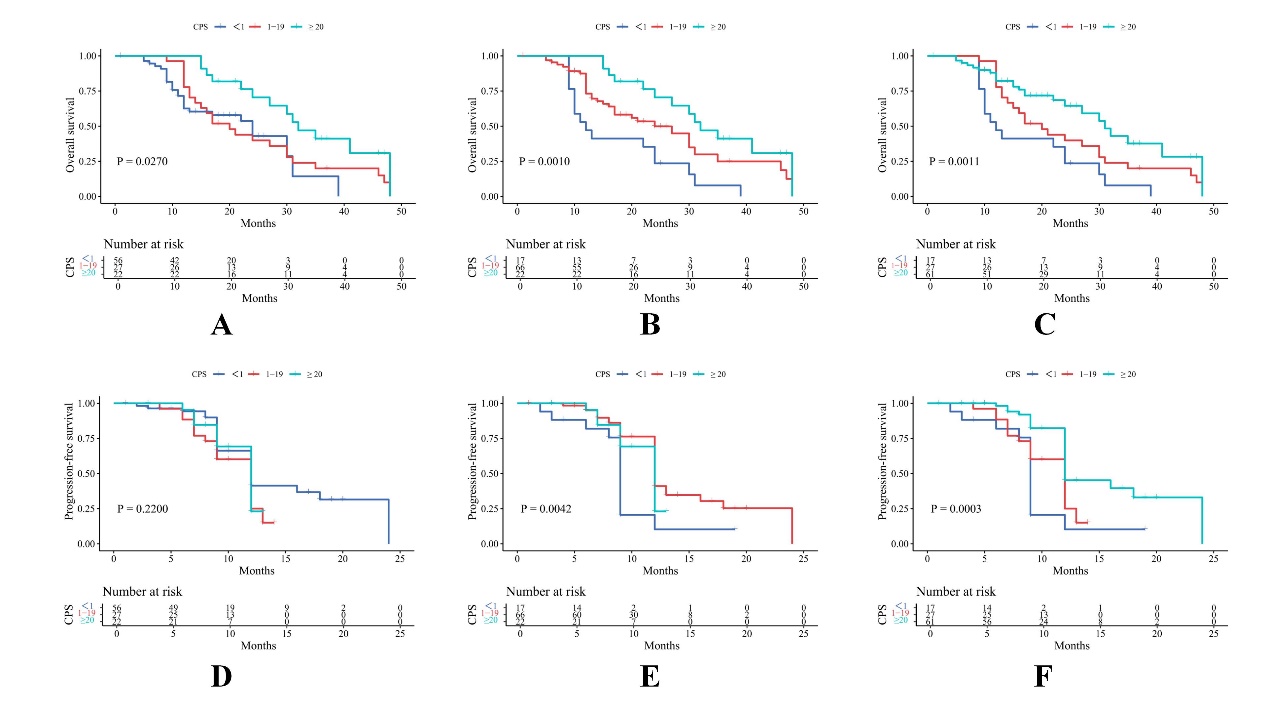


Supplementary Figure 3. Missing CPS data were addressed using multiple imputation with five imputed datasets, followed by a re-analysis of OS to evaluate the robustness of the findings.


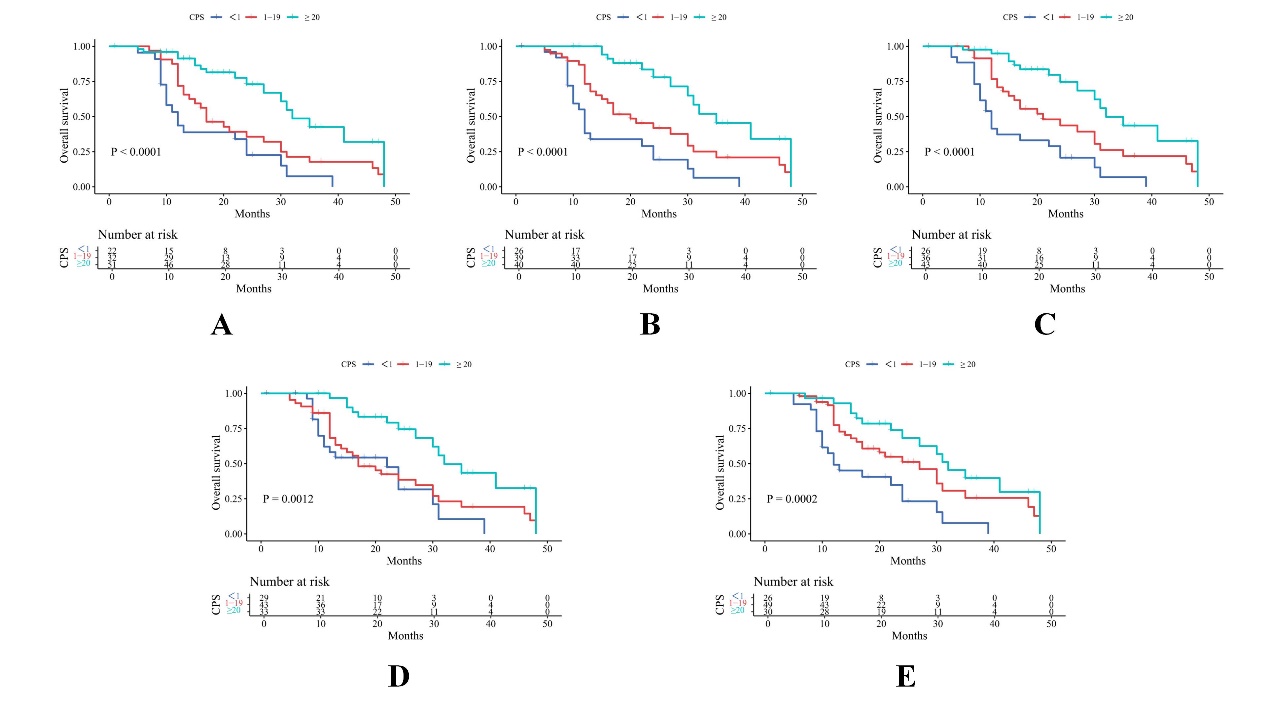


Supplementary Figure 4. Missing CPS data were addressed using multiple imputation with five imputed datasets, followed by a re-analysis of PFS to evaluate the robustness of the findings.


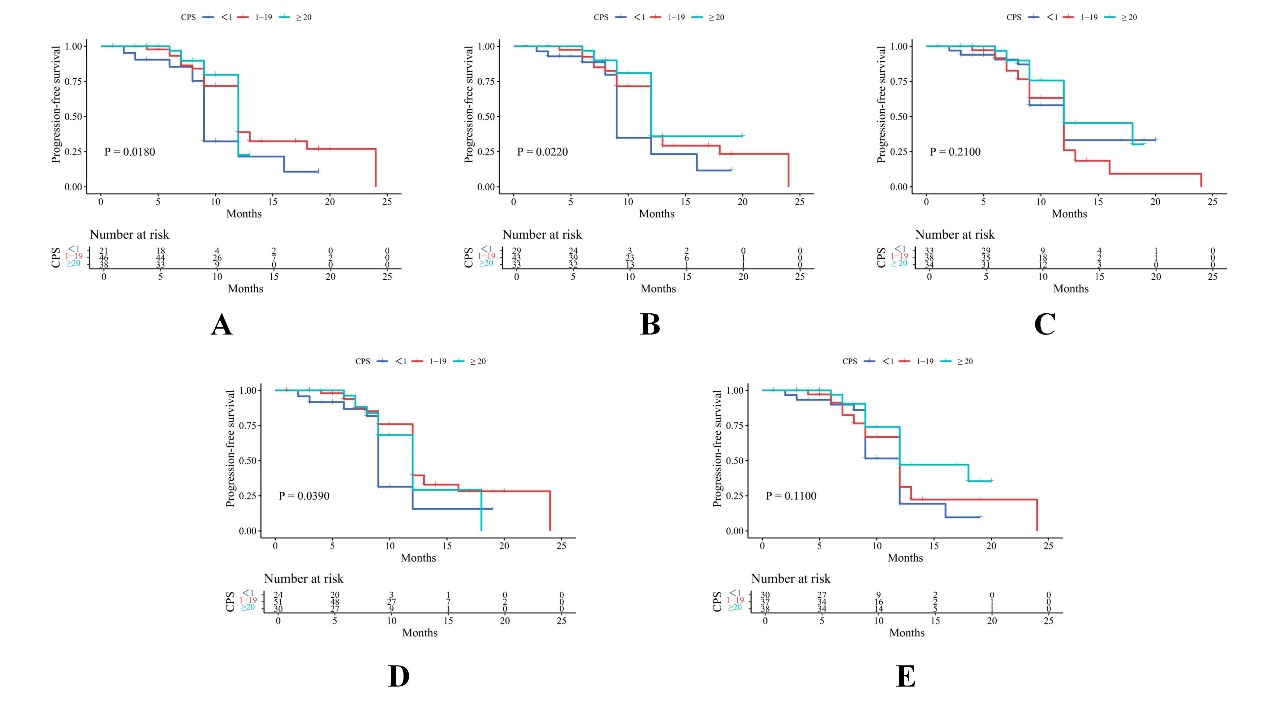


Supplementary Table 2. Univariate and Multivariable analysis of progression-free survival in 105 cases of R/M HNSCC

|  |  | Univariate | | | Multivariable | | |
| --- | --- | --- | --- | --- | --- | --- | --- |
|  |  | HR | 95% CI | P. value | HR | 95% CI | P. value |
| Age | ≥65 | 1 |  |  |  |  |  |
|  | ＜65 | 2.187 | 1.077-4.440 | 0.030 | 1.881 | 0.865-4.087 | 0.111 |
| **Gender** | **male** | **1** |  |  | **1** |  |  |
|  | **female** | **0.497** | **0.306-0.807** | **0.005** | **0.543** | **0.326-0.906** | **0.019** |
| **Cycle** | **＜5** | **1** |  |  | **1** |  |  |
|  | **≥ 5** | **0.411** | **0.214-0.787** | **0.007** | **0.401** | **0.197-0.816** | **0.012** |
| **Therapeutic Modality** | **IT alone** | **1** |  |  | **1** |  |  |
|  | **combination** | **0.457** | **0.282-0.740** | **0.001** | **0.372** | **0.223-0.619** | **0.002** |
| **ECOG** | **0** | **1** |  |  | **1** |  |  |
|  | **1** | **2.451** | **1.052-5.711** | **0.038** | **3.117** | **1.313-7.403** | **0.01** |
|  | **2** | **8.885** | **3.119-25.307** | **0.001** | **9.246** | **3.091-27.655** | **＜0.0001** |
| M stage | M1 | 1 |  |  |  |  |  |
|  | M0 | 0.958 | 0.448-2.052 | 0.914 |  |  |  |
| subsequent radiotherapy | Yes | 1 |  |  |  |  |  |
|  | No | 0.697 | 0.209-2.325 | 0.558 |  |  |  |
| CPS | ＜1 | 1 |  |  |  |  |  |
|  | 1≤ CPS＜20 | 1.451 | 0.903-2.331 | 0.124 |  |  |  |
|  | ≥20 | 1.095 | 0.956-1.254 | 0.189 |  |  |  |
| immunotherapy | imported drugs | 1 |  |  |  |  |  |
|  | domestic drugs | 0.847 | 0.358-2.006 | 0.706 |  |  |  |
| **Adverse reactions** | **1-2** | **1** |  |  | **1** |  |  |
|  | **≥3** | **7.606** | **3.084-18.76** | **＜0.0001** | **8.724** | **3.356-22.68** | **0.0007** |
| hematotoxicity | Yes | 1 |  |  |  |  |  |
|  | NO | 0.543 | 0.118-1.547 | 0.261 |  |  |  |
| Combined  chemotherapy | Paclitaxel based | 1 |  |  |  |  |  |
|  | Gemcitabine based | 0.559 | 0.197-1.817 | 0.365 |  |  |  |

Supplementary Table 3. Univariate and Multivariable analysis of overall survival in 105 cases of R/M HNSCC

|  |  | Univariate | | | Multivariable | | |
| --- | --- | --- | --- | --- | --- | --- | --- |
|  |  | HR | 95% CI | P. value | HR | 95% CI | P. value |
| Age | ≥65 | 1 |  |  |  |  |  |
|  | ＜65 | 0.663 | 0.201-2.191 | 0.500 |  |  |  |
| Gender | male | 1 |  |  |  |  |  |
|  | femal | 0.691 | 0.240-1.989 | 0.493 |  |  |  |
| **Cycle** | **＜5** | **1** |  |  | **1** |  |  |
|  | **≥ 5** | **14.44** | **1.658-25.49** | **0.001** | **2.388** | **1.061-5.327** | **0.035** |
| Therapeutic Modality | IT alone | 1 |  |  | 1 |  |  |
|  | combination | 8.889 | 1.432-13.4 | 0.0036 | 1.573 | 0.835-2.964 | 0.161 |
| ECOG | 0 | 1 |  |  |  |  |  |
|  | 1 | 1.23 | 0.591-2.572 | 0.582 |  |  |  |
|  | 2 | 1.44 | 0.520-4.001 | 0.496 |  |  |  |
| M stage | M1 | 1 |  |  |  |  |  |
|  | M0 | 0.467 | 0.148-1.476 | 0.195 |  |  |  |
| subsequent radiotherapy | Yes | 1 |  |  |  |  |  |
|  | No | 0.566 | 0.168-1.905 | 0.358 |  |  |  |
| CPS | ＜1 | 1 |  |  |  |  |  |
|  | 1≤ CPS＜20 | 1.33 | 0.8-2.19 | 0.268 |  |  |  |
|  | ≥20 | 1.28 | 0.9-1.81 | 0.169 |  |  |  |
| immunotherapy | imported drugs | 1 |  |  | 1 |  |  |
|  | domestic drug | 0.813 | 0.288-2.300 | 0.697 |  |  |  |
| Adverse reactions | 1-2 | 1 |  |  | 1 |  |  |
|  | ≥3 | 0.470 | 0.151-1.458 | 0.191 |  |  |  |
| hematotoxicity | Yes | 1 |  |  | 1 |  |  |
|  | NO | 0.258 | 0.073-0.911 | 0.0354 | 0.498 | 0.112-2.208 | 0.359 |
| Combined  chemotherapy | Paclitaxel based | 1 |  |  |  |  |  |
|  | Gemcitabine based | 0.506 | 0.227-1.129 | 0.096 |  |  |  |

Supplementary Figure 5. Heatmap representing pearson correlation coefficients for Cycles, Adverse reactions, OS, and PFS


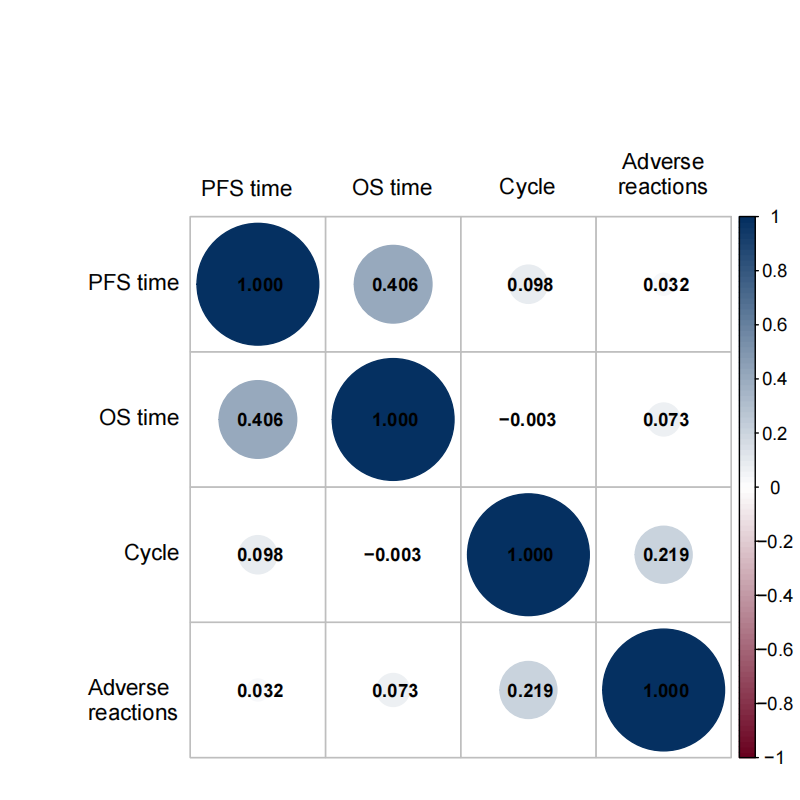


| Factor | PFS time | OS time | Cycle | Adverse reactions |
| --- | --- | --- | --- | --- |
| PFS time | 1 | 0.4064 | 0.0984 | 0.0323 |
| OS time | 0.4063 | 1 | -0.0032 | 0.0731 |
| Cycle | 0.0984 | -0.0032 | 1 | 0.2188 |
| Adverse reactions | 0.0323 | 0.0731 | 0.2188 | 1 |

Supplementary Table 4. Comparison of therapeutic outcomes across different treatment cycles

| **Cycle ≥ 5 (n = 55)** | | | **Cycle ＜ 5 (n = 50)** | | |
| --- | --- | --- | --- | --- | --- |
| CR | n = 10 | ORR = 43.63% | CR | n = 4 | ORR = 30.00% |
| PR | n = 14 |  | PR | n = 11 |  |
| SD | n = 14 | DCR = 69.09% | SD | n = 12 | DCR = 54.00% |
| PD | n = 17 |  | PD | n = 23 |  |

Supplementary Table 5. Adverse reactions

| Adverse Reaction Types | 1~2 grade | ≥3 grade |
| --- | --- | --- |
| Total incidence rate | 50/105 (47.62%) | 10/105 (9.52%) |
| Nausea and vomiting | 14/105 (13.33%) | 0 |
| Fatigue | 22/105 (20.95%) | 0 |
| Weight loss | 18/105 (17.14%) | 0 |
| Dermatitis | 15/105 (14.29%) | 2/105 (1.90%) |
| Hematologic toxicity | 48/105 (45.71%) | 9/105 (8.57%) |
| Cardiotoxicity | 3/105 (2.86%) | 0 |
| Pulmonary toxicity | 2/105 (1.90%) | 0 |
| Hepatotoxicity | 10/105 (9.52%) | 0 |
| Nephrotoxicity | 7/105 (6.67%) | 0 |
| Hypothyroidism | 15/105 (14.29%) | 0 |
| Pancreatic toxicity | 1/105 (0.95%) | 0 |

Supplementary Figure 6. Peripheral blood biomarker-based survival analysis: Dynamic changes in B lymphocytes, CD3^+^ T cells, CD4^+^ T cells, CD8^+^ T cells, NK cells, NLR, and PLR were collected; based on the baseline cutoff values of each marker, the expression levels at baseline, 6w pt, and 12w pt were categorized into two groups; subsequently, comparisons of OS and PFS were conducted; these markers exhibited no statistically significant differences.


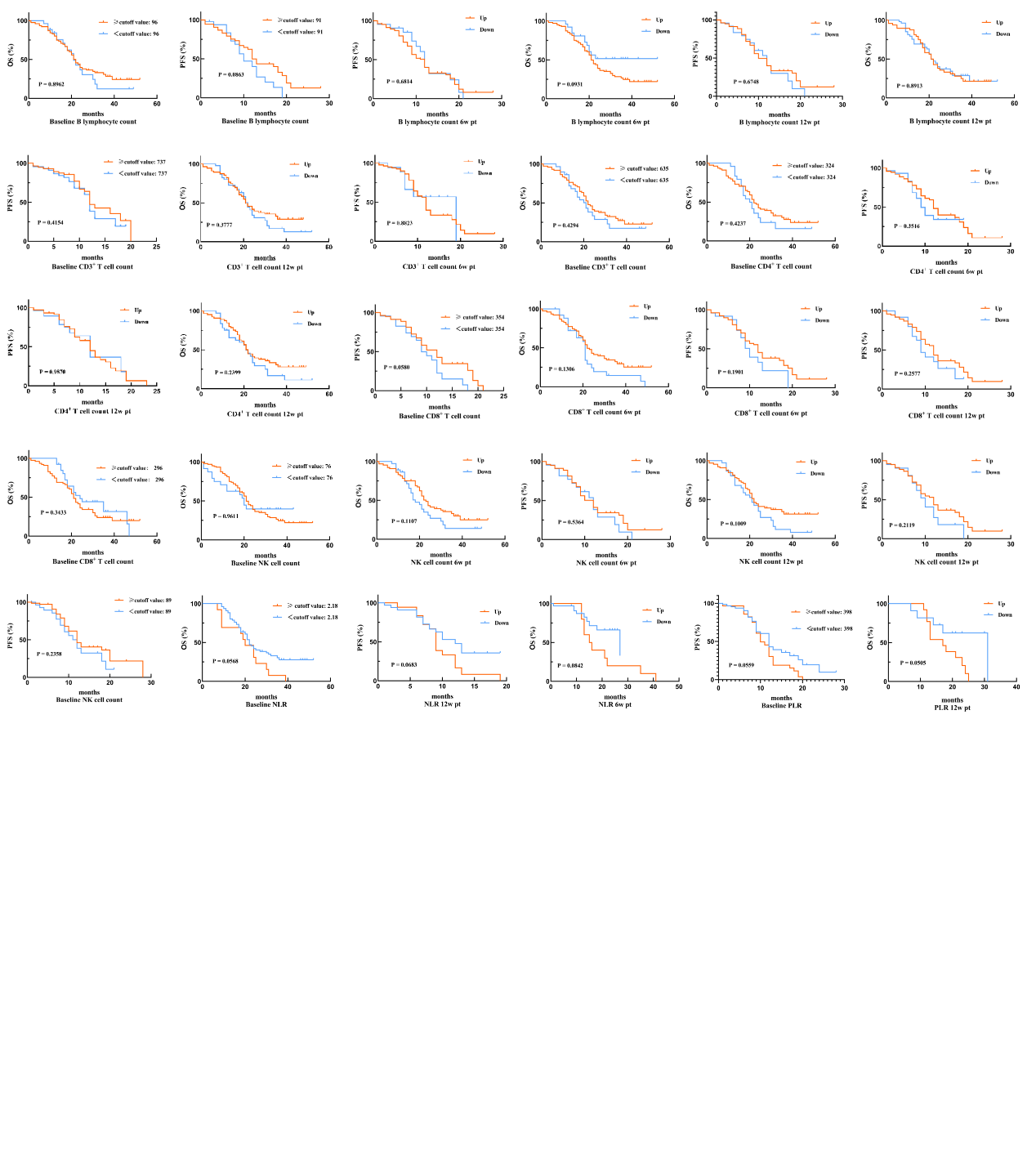


Supplementary Figure 7. A B: LASSO regression analysis; C: The markers incorporated into the model along with their corresponding correlation coefficients.


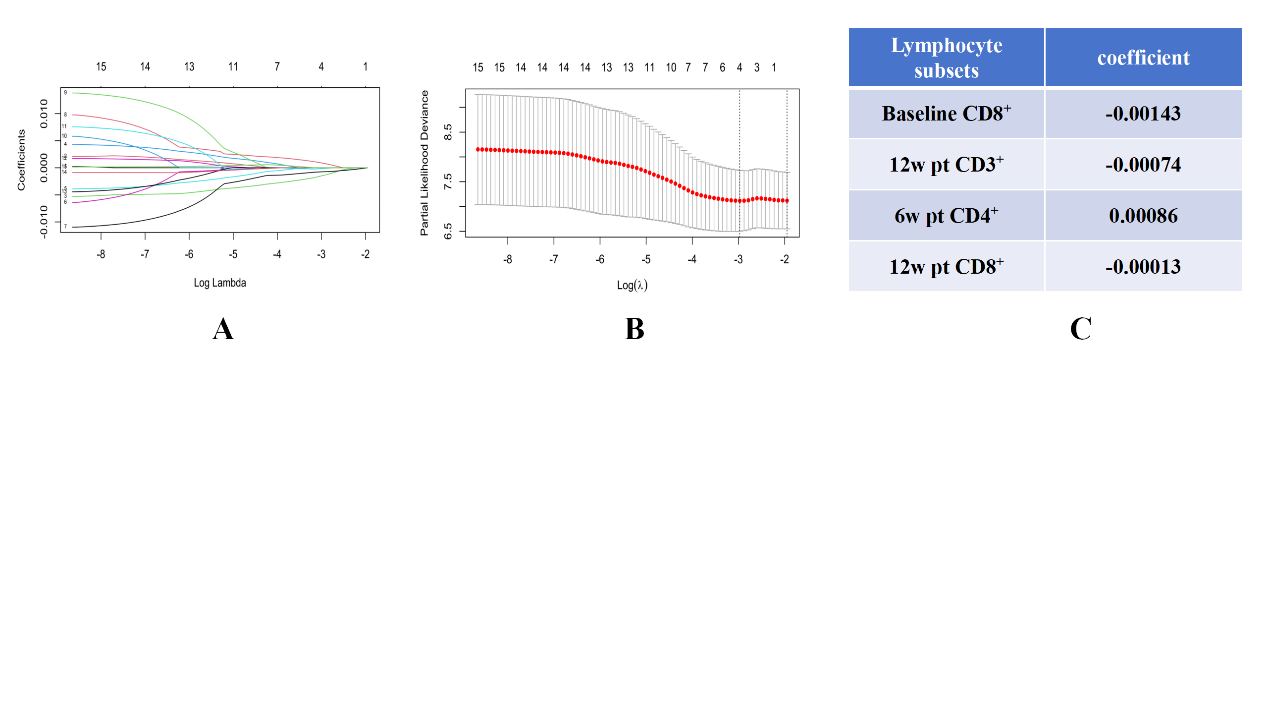

Supplement: Supplementary file 1 [file DataSheet1.docx]
